# Case Report: Macrodystrophia lipomatosa – Illustration of two cases

**DOI:** 10.4103/0971-3026.43844

**Published:** 2008-11

**Authors:** V. Singla, V Virmani, P Tuli, P Singh, N Khandelwal

**Affiliations:** Department of Radiodiagnosis, Postgraduate Institute of Medical Education and Research, Chandigarh, India; * Department of Plastic surgery, Postgraduate Institute of Medical Education and Research, Chandigarh, India

**Keywords:** Macrodystrophia lipomatosa, macrodactyly, imaging

## Abstract

Macrodystrophia lipomatosa is a rare cause of congenital macrodactyly, characterized by progressive proliferation of all mesenchymal elements, with a disproportionate increase in fibroadipose tissue. This developmental anomaly is reportedly more common in the foot than in the hand, with a predilection for the plantar and median nerve distribution. We present two cases of MDL of the hand, one of which had an unusual nerve territory distribution, making clinical diagnosis difficult. Preoperative diagnosis was however made on the basis of radiography and MRI and was later confirmed on surgery.

Macrodystrophia lipomatosa (MDL) presents as localized gigantism of the hand or foot and comes to clinical attention because of cosmetic reasons, mechanical problems secondary to degenerative joint disease, or development of neurovascular compression due to large osteophytes.[1] Radiological investigations, especially MRI, help to make a definitive diagnosis noninvasively and to differentiate it from other causes of macrodactyly. We review the clinical and radiological features of MDL by describing two cases, both of which had involvement of the upper limb digits. In the first case, the pathology was confined to the thumb and index finger, while in the second case, the ring and little finger were involved, which is rarely reported in the literature.

## Case Reports

### Case 1

**Figure 1:**
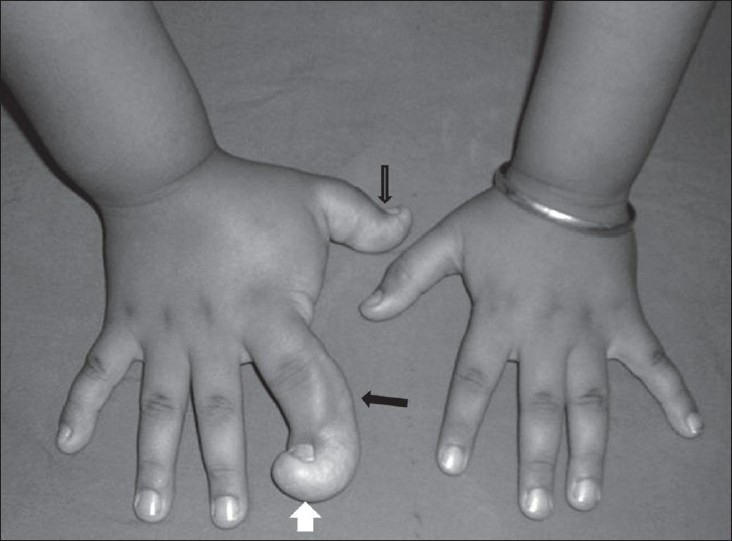
Case 1: Clinical photograph of both hands demonstrates the macrodactyly involving the thumb and index finger (black arrows) of the right hand and the dorsal angulation of the index finger (white arrow)

**Figure 2:**
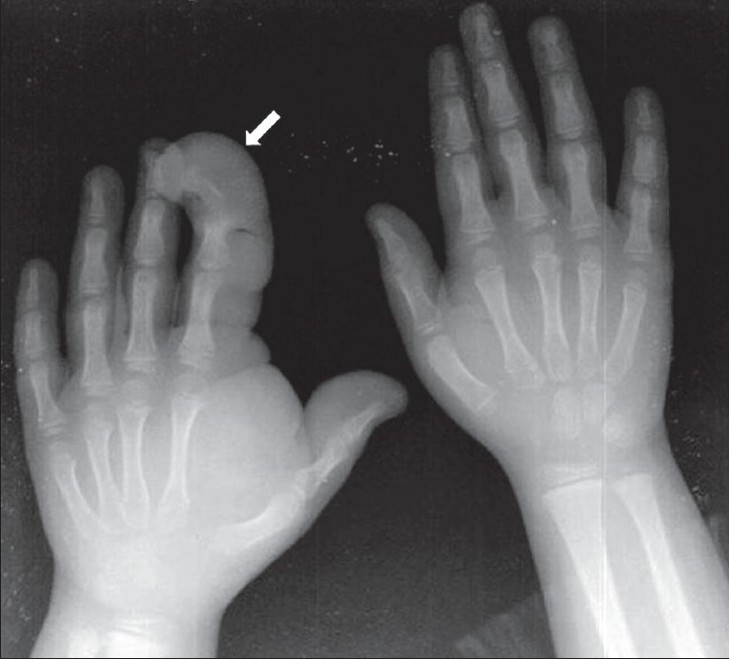
Case 1: Plain radiograph of both hands shows soft tissue swelling (arrow) on the palmar aspect of the affected fingers; the distal end is predominantly affected, with splaying of the phalanges

**Figure 3:**
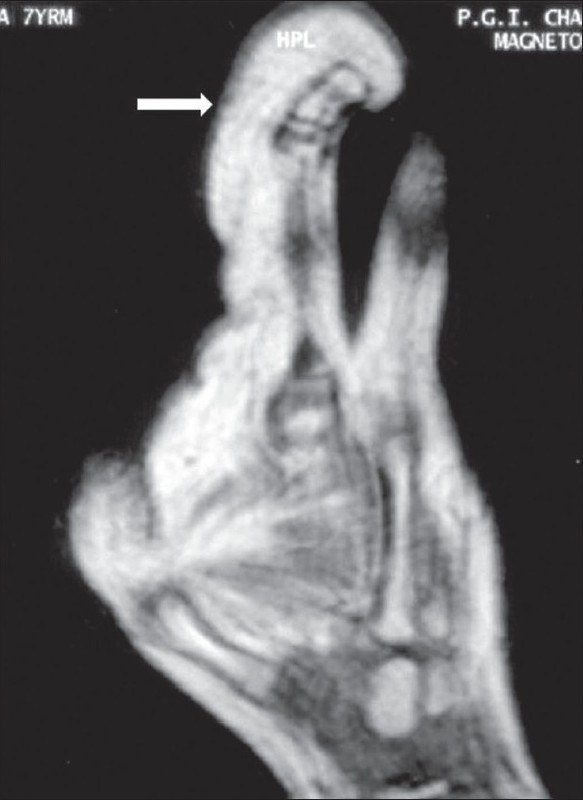
Case 1: Sagittal T1W MRI image reveals proliferation of fatty tissue (arrows) on the volar aspect of the index finger with signal intensity similar to that of subcutaneous fat; there is dorsal angulation of the affected digit

A 7-year-old, right-hand-dominant male child presented with a progressive disproportionate enlargement of the thumb and index finger of his right hand since 1 year of age. The patient denied having any pain or neurovascular symptoms and there was no family history of extremity gigantism. On physical examination, a nontender, soft tissue mass was palpable on the volar aspect of the enlarged fingers and there was dorsal angulation of the index finger [Figure 1]. There were no overlying cutaneous changes, pitting edema, or bruit. The patient was able to use the hand well and had an adequate grip. A plain radiograph demonstrated soft tissue swelling along the volar aspect of the thumb and index finger, an enlarged distal phalanx of the index finger, and osteoarthritic changes in the distal interphalangeal joint [Figure 2]. An MRI with a 1.5-T unit demonstrated increased fatty tissue along the palmar aspect of the right index finger and thumb, extending up to the thenar eminence. It was seen as a hyperintense area on T1W images with intermediate signal on T2W images [Figure 3]. The underlying bones revealed normal signal intensity and intact periosteum.

### Case 2

**Figure 4:**
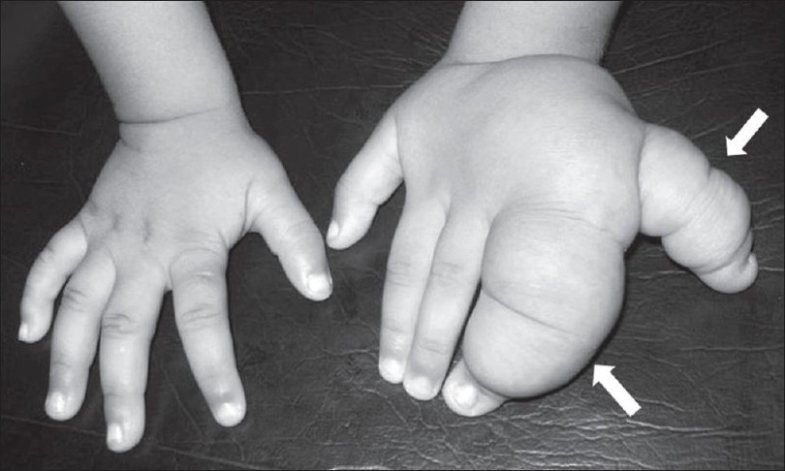
Case 2: A clinical photograph of both hands shows enlarged ring and little fingers (arrows) of the left hand as compared to the normal right hand

**Figure 5:**
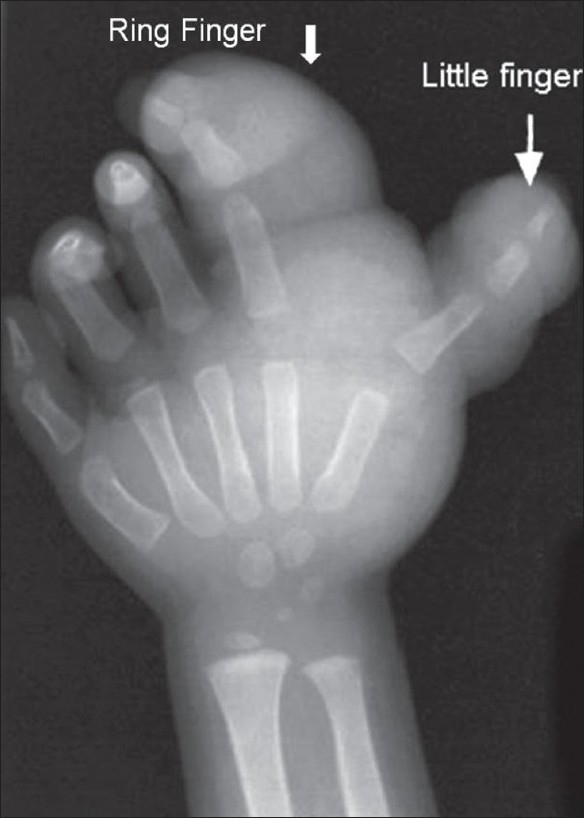
Case 2: Plain radiograph of the left hand shows soft tissue swelling (arrows) causing enlargement of the ring and little fingers, along with dorsal angulation of the former and lateral angulation of the latter

**Figure 6:**
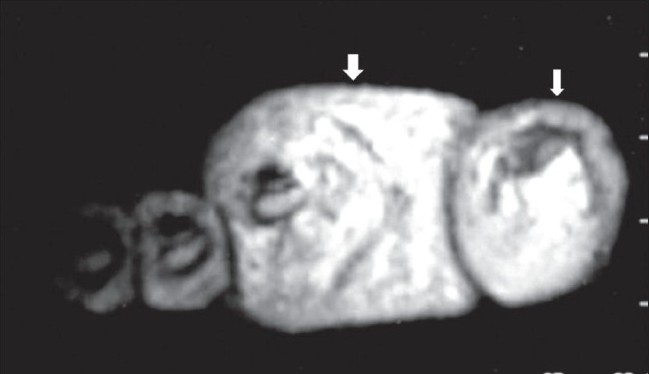
Case 2: Axial T2W MRI image shows fat-intensity soft tissue (arrow) surrounding these digits

**Figure 7:**
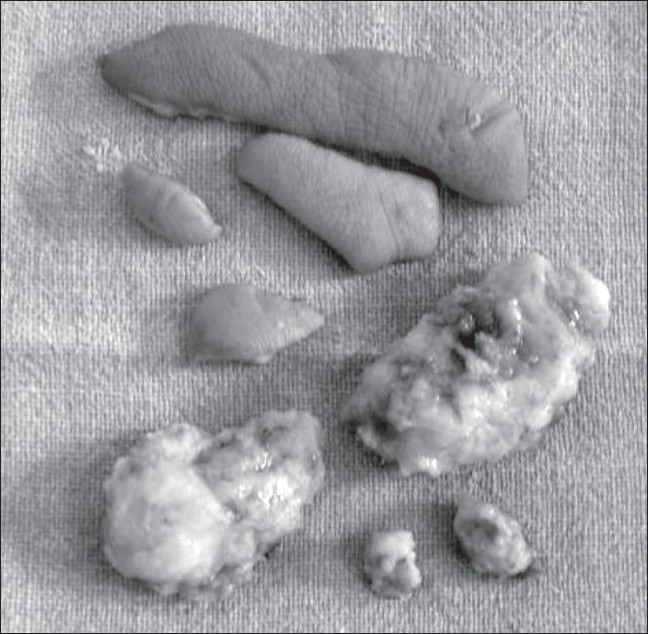
Case 2: Photograph shows the excised predominantly adipose tissue

A 2-year-old right-hand-dominant male child presented with progressive enlargement and swelling of both dorsal and palmar aspects of the left fourth and fifth digits since birth [Figure 4]. No tenderness, pitting edema, bruit, or skin changes were present and the movements of the digits were normal. A radiograph revealed disproportionately enlarged left ring and little fingers; there was soft tissue swelling, along with dorsal angulation of the fourth finger and lateral angulation of the fifth finger [Figure 5]. MRI depicted fatty tissue proliferation around the left fourth and fifth digits, which was more along the dorsal aspect [Figure 6]. There was no medullary or periosteal abnormality. A debulking procedure was performed and the fingers were reconstructed. Extraction of fat [Figure 7] was done through an incision placed over the dorsum of the ring finger extending to the hypothenar eminence, care being taken to spare the extensor tendons. Pathological examination of the material revealed abundant adipose tissue with some interspersed fibrous tissue.

## Discussion

MDL is an uncommon congenital, nonhereditary, localized gigantism involving the digits or extremities. This term was first used by Feriz in 1925 to describe unilateral overgrowth of the lower limb.[2] Though the exact etiology of MDL is not known, various hypotheses exist, including alteration of somatic cells during limb bud development and disturbed fetal circulation.[3]

MDL usually presents at birth and may be associated with anomalies like syndactyly, polydactyly, brachydactyly, or clinodactyly.[4] Association with small osseous protuberances, which resemble osteochondromas and lipomas, in other parts of the body has also been reported.[5] The disease is almost always unilateral, with an equal incidence in males and females.[6] The growth velocity may differ from digit to digit and the abnormal growth usually ceases at puberty. The lower limb is more frequently involved than the upper limb. The abnormal area is usually along a specific sclerotome. The second and third digits of the hands and feet are most frequently involved, corresponding to the median nerve and medial plantar nerve supply in the upper and lower limbs, respectively.[7] Involvement of the ulnar nerve distribution, as seen in our second case, is extremely unusual.[4] The soft tissue overgrowth is most marked at the distal ends of the digits on the volar aspect and results in dorsal angulation of the involved digit.

Different imaging modalities, such as plain radiography, USG, CT scan, and MRI, have a role in the evaluation of MDL. Plain radiography reveals hypertrophy of soft tissue and bone, with translucencies in the soft tissue due to increased adipose tissue. The phalanges, especially the distal phalanx, are elongated, broad, and splayed, sometimes giving rise to a mushroom-like appearance. Secondary osteoarthritic changes like joint space narrowing, subchondral cysts, and osteophytes often develop in adolescence or early adulthood.[1] Both USG and CT scan can be used to demonstrate the proliferation of fat along the nerve territory.[8,9] MRI easily demonstrates the excess fibrofatty tissue, which has signal characteristics similar to subcutaneous fat, i.e., high signal on T1W, intermediate signal on T2W, and low signal on fat-suppressed sequences. The fat in MDL is not encapsulated. The fibrous strands within the fatty tissue are seen as low-signal-intensity linear strands on T1W images.

The differential diagnoses of MDL and macrodactyly include neurofibromatosis, hemangiomatosis, lymphangiomatosis, Proteus syndrome, and fibrolipomatous hamartomas. Neurofibromas show marked hyperintensity on T2W images and are seen in close relation to nerves. A positive family history, presence of cutaneous lesions, and bilaterality favor neurofibromatosis, while hypertrophy along a nerve territory, unilaterality, and demonstration of fat within the nerve sheath on MRI favor MDL.[10] Lymphangiomas are hyperintense to muscle on T1W and hyperintense to fat on T2W images. Clinically, diffuse swelling and pitting edema are found.[8] In hemangiomatosis, a bruit may be palpable clinically and, on MRI, long TR/TE sequences show a septate configuration of high-signal-intensity channels, corresponding to the vascular channels and fibrous strands found in hemangiomas. Osseous growth is not seen in both lymphangiomatosis and hemangiomatosis.[8] Proteus syndrome presenting with hemihypertrophy may simulate MDL, but associated abnormalities like calvarial changes, pulmonary cysts, pigmented nevi, and intra-abdominal lipomas help to arrive at the correct diagnosis. Some consider MDL to be a localized form of Proteus syndrome. Fibrolipomatous hamartoma (FLH) of nerve is a rare tumor-like condition in which mature fat infiltrates the neural sheath, with the majority of the lesions occurring in the median nerve. Pathologically, in FLH, the deposition of fat is within the nerve sheath, while in MDL it occurs throughout the involved part of the digits/extremity. However, MDL may be an associated feature of FLH in as much as 30–66% of cases.[11] FLH may show a speckled appearance on MR, correlating with its histologically known architecture, i.e., neural fascicles separated by fat and connective tissue.

In conclusion, determination of the cause of macrodactyly is clinically difficult due to the many possible etiologies. However, appropriate imaging, particularly with MRI, can make the determination of the underlying process easier and can be of great help in arriving at a correct diagnosis. Imaging helps in differentiating MDL from other causes of localized hemihypertrophy, which have different prognoses, complications, and treatment.
